# Chronic Effects of Different Resistance Training Exercise Orders on Flexibility in Elite Judo Athletes

**DOI:** 10.2478/hukin-2014-0015

**Published:** 2014-04-09

**Authors:** Alam R. Saraiva, Victor M. Reis, Pablo B. Costa, Claudio M. Bentes, Gabriel V. Costa e Silva, Jefferson S. Novaes

**Affiliations:** 1 Federal University of Rio de Janeiro. Physical Education - Graduate Program. Rio de Janeiro, RJ – BRAZIL.; 2 Research Center for Sport, Health, and Human Development (CIDESD), University of Trás-os-Montes and Alto Douro (UTAD), Vila Real, PORTUGAL.; 3 Human Performance Laboratory, Department of Kinesiology, California State University – San Bernardino, San Bernardino, CA, USA.

**Keywords:** strength training, static stretching, martial arts, exercise sequence

## Abstract

The aim of this study was to examine the effects of twelve weeks of resistance training with different exercise orders (upper limbs and lower limbs vs. lower limbs and upper limbs) on flexibility levels in elite judo athletes. Thirty-nine male athletes were randomly divided into 3 groups as follows: G1 (n = 13), G2 (n = 13), and CG (n = 13). The flexibility was assessed on 8 joint movements: shoulder flexion and shoulder extension, shoulder abduction and shoulder adduction, trunk flexion and trunk extension, and hip flexion and hip extension. Two-way repeated measures ANOVAs (time [pre-experimental vs. post-experimental] × group [G1 vs. G2 vs. CG]) were used to compare the differences between pre- and post-test situations and the differences among groups. The results from the within-group (pre vs. post) comparisons demonstrated significant increases (p < 0.05) in the range of motion of 3.93 and 5.96% for G1 and G2 training groups, respectively, in all joints. No significant changes (p > 0.05) were observed for the CG. The results from the between-group comparisons demonstrated no significant differences (p > 0.05) in the range of motion between G1post vs. G2post (1.15%). Although both exercise orders (from upper to lower limbs and from lower to upper limbs) increased flexibility, no significant variations were observed between the different exercise orders. Nevertheless, these findings demonstrate that flexibility gains could be obtained with a resistance training program, and thus, more time can be devoted to sports-specific judo training.

## Introduction

The martial art and combat sport of judo has been an Olympic event since 1964 (Nishime, 2007) being represented by 187 countries in the International Judo Federation (IJF). Accordingly, judo is one of the most popular sports in the world (Amtmann and Cotton, 2005). The sport of judo entails throwing the opponents onto their backs using different techniques (e.g., leg, ashi-waza, te-waza, koshi-waza, and sutemi-waza) or to score during groundwork combat (grappling) using immobilizations (ossae-waza, shimewaza), or elbow joint locks (kansetsu-waza). This combat sport involves great neuromuscular demand, suggesting that a good level of physical fitness in strength and flexibility seems crucial to its competitors (Fukuda et al., 2011). For instance, competitors are matched by weight, hence, higher overall body strength is an important advantage (Blais and Trilles, 2006). Each bout normally lasts up to 5 minutes and is characterized by high intensity intermittent exercise similar to wrestling; however, chokes and joint lock manipulations are allowed. In order to perform these techniques, the participants overload their muscles and joints, especially those associated with the shoulder, trunk and the hip movements (Franchini et al., 2013; Fukuda et al., 2011; Imamura et al., 2006).

Despite the American College of Sports Medicine (2011) recommendations that resistance training and stretching exercises should be used in training programs to improve the major components of physical fitness and health in individuals of all ages, scientific information regarding the resistance training responses in judo athletes is scarce. It is known that adequate strength and flexibility levels can provide significant benefits in functional and sports performance. However, physical fitness level, gender and age can affect strength and flexibility performance. Accordingly, resistance training improves strength performance and the range of motion in many joints demonstrating resistance training may be an appealing method of conditioning to increase strength and flexibility concomitantly (Morton et al., 2011; Simao et al., 2011). Nevertheless, training studies investigating the chronic effects of resistance training on flexibility have used different training methods and other resistance training methodological variables. Recently, Costa et al. (2013) and Costa et al. (2009) reported that pre-exercise flexibility training might cause negative acute effects on strength while it might not reduce the risk of injuries. Considering that judo athletes may have little time for additional training and because flexibility training can impair strength performance, resistance training appears to be a potential strategy to improve both physical fitness components (strength and flexibility) (Santos et al., 2010; Simao et al., 2011; Souza et al., 2013).

Studies have compared the effects of different resistance training methods separately (Fatouros et al., 2006; Kim et al., 2011; Morton et al., 2011) or in association with stretching exercises (Fatouros et al., 2006; Monteiro et al., 2008; Simao et al., 2011; Souza et al., 2013) on flexibility. For example, Monteiro et al. (2008) reported significant improvements in flexibility in sedentary middle-age men and women after ten weeks of resistance circuit training. Studies that analyzed flexibility after manipulating different resistance training variables have generally demonstrated significant gains in flexibility in other populations as well (Fatouros et al., 2006; Kim et al., 2011; Santos et al., 2010). For instance, Fatouros et al. (2005) and Fatouros et al. (2006) compared different resistance training intensities (40 vs. 80% of 1RM) examining their effects on flexibility levels and reported increases in the range of motion. In addition, different movement velocities (slow × super-fast) have also been reported to increase the range of motion (Kim et al., 2011).

Studies focusing on the effects of exercise order on the range of motion are still scarce. To our knowledge, the only study that analyzed resistance training responses with different exercise orders on flexibility was Santos et al. (2010). Twenty-four young sedentary women were examined after eight weeks of resistance training with different exercise order groups and showed significant increases in the range of motion of the trunk and shoulder joints. Although, Santos et al. (2010) changed the resistance training exercise order by alternating upper and lower body exercises or different muscle actions (agonist-antagonist), we are unaware of studies investigating the effects of different exercise orders on flexibility in judo athletes. However, the resistance training responses on flexibility levels need to be further investigated, especially its implication in judo athletes. These results will help coaches to plan the best strategies for athlete’s conditioning and determine whether stretching exercises will be included or not after training sessions. Therefore, the aim of this study was to examine the effects of twelve weeks of resistance training with different exercise orders (upper limbs progressing to lower limbs vs. lower limbs progressing to upper limbs) on flexibility levels in elite level judo athletes.

## Material and Methods

### Participants

Thirty-nine professional judo male athletes with at least five years of competitive experience and who were training to compete in the upcoming Brazilian Judo National Championships volunteered for the study. Participants were divided into 3 groups as follows: G1 (n = 13), G2 (n = 13), and CG (n = 13) (Table 1). Prior to subject participation and data collection, all subjects answered the Physical Activity Readiness Questionnaire and signed an informed consent form according to the Declaration of Helsinki. All athletes were familiar with the resistance training exercises, and did not have any recent history of upper or lower body injury. This research project was approved by the University Ethics Committee under the protocol number CAAE: 0070.412.000-11.

### Measures

The testing measurements for this study were completed during the preparation for the Brazilian Judo National Championships. Before beginning the 12-week training program, athletes were randomly assigned to 1 of 3 groups: experimental group 1 (G1), experimental group 2 (G2), or a control group (CG). The G1 exercise order was bench press (BP), lat pull-down (LA), shoulder press (SP), and arm curl (AC) for the upper body; and squat (SQ), leg press (LP), leg extension (LE), and leg curl (LC) for the lower body. For G2, the order was SQ, LP, LE, and LC for the lower body; and BP, LA, SP, and AC for the upper body. The CG did not take part in the resistance training program. Initial flexibility was assessed 48 to 72 hours after the initial 10RM testing procedure and before the resistance training program commenced. At the end of the 12-week training period, flexibility was reassessed 48 hours after the final training session. Training included 3 sessions per week, performed 24 h apart, for a total of 36 sessions. The athletes from the 2 intervention groups completed all training sessions.

### Flexibility Measurements

Flexibility was assessed on eight joint motions: shoulder flexion (SF) and shoulder extension (SE), shoulder abduction (SAB) and shoulder adduction (SAD), trunk flexion (TF) and trunk extension (TE), and hip flexion (HF) and hip extension (HE). Except for trunk movements, all assessments were collected on the right side of the body. The TF, TE, and SAD were performed in the orthostatic position. The SAB movement was performed while the subject was seated. The SF and SE were assessed on a trolley to limit compensatory movement. To assess flexibility, the investigator adjusted the subject’s body to the pain threshold or anatomical limitation. The measurements were taken using a Lafayette Goniometer (Sammons Preston Rolyan 7514), according to the procedures described by Norkin and White (2003). The repeatability of the flexibility tests was determined using the intra-class correlation coefficient (ICC). Excellent day-to-day reliability (test and re-test) was shown for each assessment (ICC ranged between 0.93 and 0.99). In addition, paired Student’s t-tests revealed no significant differences between the two days for any of the flexibility measurements.

### Ten Repetition Maximum (10RM) Strength Testing

To obtain reliable 10 RM loads, data were assessed during two non-consecutive days in the following exercise sequences: BP, LA, SP, and AC for upper body exercises; and SQ, LP, LE, and LC for lower body exercises. During the 10RM test, each subject performed a maximum of three 1RM attempts for each exercise with 5-minute rest intervals between each attempt. After the 10RM load in a specific exercise was determined, a rest period of at least 10 minutes was allowed before attempting the 10RM for the next exercise. The 10RM tests were repeated after 48 hours to determine test-retest reliability. Excellent day-to-day reliability was shown for each exercise (ICC ranged between 0.97 and 0.99). In addition, paired Student’s t-tests revealed no significant differences between the two days for any of the 10RM tests. The highest load in each test was determined to be the 10RM. The 10RM testing protocol has been previously described by Simão et al. (2005).

### Exercise Sessions

Before the beginning of each training session, a warm-up that consisted of 12 repetitions at 50% of the 10RM load of the first exercise was performed. The training groups (G1 and G2) performed 3 sets of 10–12 repetitions each at the 10RM load for each exercise. The G1 exercise order progressed from the upper to lower body. For G2, the order progressed from the lower to upper body. The CG did not perform any resistance training program. When the upper limit of 12 repetitions for a given exercise was exceeded, the load for that particular exercise was increased by 5% before continuing with the subsequent 10–12 RM set. During all of the resistance training sessions, the athletes were asked not to perform the Valsalva manoeuver. All sets were performed until concentric failure or an undesirable change in lifting mechanics was observed. Pauses were not allowed between the concentric and eccentric phases, and all of the movements were performed at moderate and controlled velocities. All athletes performed the exercises bilaterally and rested for 2-minute intervals between sets and between exercises. Although the CG did not perform any resistance training, all participants continued to take part in their judo-specific training during the course of the study. The judo-specific training program was similar to those described by Fukuda et al. (2013), Costa et al. (2011), and Baudry et al. (2009). Judo-specific training consisted of eight training sessions per week with each session lasting approximately two hours.

### Statistical Analyses

Normality and homoscedastic Shapiro-Wilk tests (with the Bartlett criterion) were performed and all variables exhibited normal homoscedasticity and distribution. Two-way repeated measures ANOVAs (time [pre-experimental vs. post-experimental] × condition [G1 vs. G2 vs. CG]) were used to compare the differences between the pre- and post-tests and the differences among groups. When appropriate, specific differences were determined using Tukey’s post hoc tests. An alpha level of p < 0.05 was considered statistically significant for all of the comparisons. SPSS software version 20.0 (SPSS Inc., USA) was used for all the statistical analyses. Effect sizes (ES) (the difference between pre-test and post-test scores divided by the pre-test standard deviation) were used to determine the magnitude of the differences observed between the pre- and post-tests (Table 3). The scale proposed by Rhea (2004) was used to classify the magnitude of effects.

## Results

The results from the within-group (prevs. post-test) comparisons demonstrated significant increases (p < 0.05) in the range of motion for the two training groups (G1_pre_ vs. G1_post_; G2_pre_ vs. G2_post_) in all joints. The results from the between-group comparisons also demonstrated significant increases (p < 0.05) in the range of motion for G1_post_ vs. CG_post_ in TF and G2_post_ vs. CG_post_ in SF (Table 2). However, no differences were observed between protocols (G1_post_ vs. G2_post_). Table 3 displays the results of the effect size analyses after 12 weeks of training.

## Discussion

The present study investigated the effects of 12 weeks of different resistance training exercise orders on flexibility in judo athletes. The results demonstrated the resistance training program increased flexibility levels in all of the joints assessed, independently of the exercise order in both training groups (G1 and G2). The range of motion gains were significantly greater when comparing G1_post_ vs. GC_post_ for the TF and G2_post_ vs. GC_post_ for the SF. Therefore, resistance training can provide significant gains in the joint range of motion in judo athletes independently of the exercise order and that gains could be different in other joints. Nevertheless, it must be emphasized that independently of exercise order, all resistance training exercises should be performed through the entire range of motion (Borman et al., 2011; Fleck and Kraemer, 2003; Garber et al., 2011). Presumably, an increase in the range of motion from stretching could also increase the range of motion in resistance training exercises.

The ES results showed moderate changes for all joints assessed in the G1 group. The G2 group showed moderate ES changes with exceptions in SE and HF, where they were small. All of the ES changes for the CG were not significant. Thus, our results indicate higher increases in flexibility of the shoulder joints for G2 and in the trunk for G1. Nevertheless, the ES after 12 weeks of resistance training were similar in all of the joints independently of exercise order. However, when comparing the ES results in all of the groups, it appears more interesting to plan a resistance training session, progressing from any exercise order based on the priority principle. Accordingly, begin the session prioritizing the most important limbs for each elite judo athlete individually. In contrast, no differences were observed between the G1_post_ and G2_post_ groups. These results demonstrate that the exercise order did not have any influence on flexibility levels. Nonetheless, the applied type of exercise may result in their increase.

Santos et al. (2010) demonstrated resistance training (8 weeks) at moderate intensity (3 sets of 10–20 reps per exercise) in two different exercise orders (4 exercises for upper limbs and 4 exercises for lower limbs vs. 8 exercises alternating upper and lower body) can increase shoulder and trunk flexibility similarly. Although no differences were found between the different exercise orders in the study of Santos et al. (2010) in sedentary women, gains in the joints’ range of motion were more significant when exercise order was used alternating upper and lower body, corroborating with our results. Therefore, their findings are in agreement with those reported by Monteiro et al. (2005). In contrast, the present study was the first to demonstrate resistance training may also increase flexibility of judo athletes. The results suggest training programs of which sessions are initiated by upper limb exercises and progress to the lower limbs provide benefits in flexibility, similarly to when the exercise order is reversed (lower limbs to upper limbs). However, the ES in both groups (G1 and G2) showed similar effects. Hence, our results could be considered similar to those reported by Santos et al. (2010), although, the sample groups were different (male athletes vs. sedentary women) and each sample had different neuromuscular features, primarily by the specificity of the judo sport demand. In addition, we examined different methodological variables of resistance training (exercise order). Therefore, according to the present study, judo athletes should begin a resistance training prioritizing the specific needs of each athlete (Bentes et al., 2012; Simao, Figueiredo, et al., 2012; Simao, Salles, et al., 2012). Future studies should investigate whether the exercise type in specific flexibility training might also affect the range of motion gains.

Although the literature is rather consistent with regard to the ability to increase flexibility through resistance training, according to the study by Nobrega et al. (2005), it might not increase flexibility in young adults. Moreover, Nobrega et al. (2005) assessed flexibility through the Flexitest (Araújo, 2004), which is a subjective measurement, where small errors in interpretation can lead to fundamental differences in results from different conditions. The present study measured flexibility by goniometry, where movements were performed at maximum amplitude, and demonstrated excellent reliability. Therefore, these are important aspects for limiting possible extrapolations and comparisons between results from the present study and those reported by Nobrega et al. (2005).

The chronic studies that examined the impact of resistance training on flexibility levels were conducted through different methodological designs and produced similar results. Accordingly, these studies investigated the effects of resistance training on the elderly (Barbosa et al., 2002; Fatouros et al., 2006; Fatouros et al., 2005), men (Morton et al., 2011; Nobrega et al., 2005), women (Kim et al., 2011; Morton et al., 2011; Simao et al., 2011), and untrained participants. This hinders the discussion of our results, since the responses may differ depending on age and gender of participants, fitness levels, training protocols, and joints examined (Santos et al., 2010). According to Fatouros et al. (2006), younger participants need to train at a higher intensity to experience the benefits of an increased range of motion. These benefits, according to Kim et al. (2011), can be obtained independent of the speed of movement of exercise during training sessions, as well as the execution in high intensity can generate neuromuscular adaptations of larger changes (Fatouros et al., 2006).

One hypothesis for the possibility of obtaining a greater range of motion with resistance training is that this mode of exercise is able to develop the tensile strength of ligaments and tendon structures, and can increase the power of contraction and muscle mass (Spirduso, 1995). In this sense, other mechanisms that have not yet been fully clarified may also justify the flexibility gains through the use of resistance training such as an increase the collagen’s turnover tax on different structures of the musculoskeletal system. Since the synthesis and degradation tax of the collagen fibers can be changed by physical activity, as a consequence of an increase of the mechanical stress applied along the longitudinal axis of the fibers, a decrease on the formation of cross-bridges is observed. The decrease on the quantity of cross-bridges, especially on the tendon, allows a better deformation of this structure (extensibility), reducing the chances of rupture, in addition to easing the transmission of the force generated by the muscles to the bones, which would lead to an increase in the movement range (Kovanen et al., 1984; Ladouceur et al., 2000). Moreover, a better force transmission, linked to specific resistance training adaptations, such as an increase of the capacity of generating muscular strength by trained muscles and the reduction of the antagonistic muscles co-activation, can cause an increase in the movement range when this is performed actively. Further studies involving longer training periods and different joints, exercise orders, and sample groups, should be conducted to extend these findings.

To our knowledge, the present study was the first to compare the effects of applying resistance training with different exercise orders on flexibility gains in elite judo athletes. Although both exercise orders (progressing from upper to lower limbs and from lower to upper limbs) increased flexibility, it was observed that gains may undergo some variations due to the manipulation of this order. Consequently, more important changes can be obtained independently of exercise order. In addition, concomitant improvements in strength and flexibility can be obtained with one mode of training, which is important because valuable time can be devoted towards sports-specific training. Based on the present results and given a training program’s time cost and the universal desire to achieve maximal physical performance in minimal time, the professionals involved in strength and conditioning should consider that resistance training might be able to increase flexibility levels in athletes and minimize the time spent in stretching allowing for more time for sports-specific training.

## Figures and Tables

**Table 1:** Baseline anthropometric characteristics (mean ± SD).

	**G1 (n = 13)**	**G2 (n = 13)**	**CG (n = 13)**
Age (years)	20.69 ± 2.36	20.23 ± 2.45	20.15 ± 1.57
Body Height (cm)	167.9 ± 0.07	169.8 ± 0.05	172.0 ± 0.06
Body Mass (kg)	71.70 ± 12.39	70.86 ± 11.59	78.36 ± 11.28
BMI	25.35 ± 3.47	24.51 ± 3.25	26.48 ± 3.04
RT Experience (Years)	5.4 ± 2.1	5.2 ± 1.7	5.3 ± 1.9
Judo Experience (Years)	6.40 ± 2.63	5.91 ± 2.24	6.14 ± 2.50

BMI = Body Mass Index, RT = Resistance Training

**Table 2:** Flexibility measurements (cm) at baseline and after 12 weeks of resistance training (mean ± SD).

	**G1 (n = 13)**		**G2 (n = 12)**		**CG (n = 13)**	

	**Baseline**	**12 weeks**	**Baseline**	**12 weeks**	**Baseline**	**12 weeks**

**SF**	178.69 ± 8.61	188.31 ± 8.79^*^	180.54 ± 6.84	189.15 ± 6.77^*†^	181.23 ± 7.61	181.69 ± 6.81
**SE**	92.31 ± 7.05	101.08 ± 8.32^*^	85.92 ± 9.90	88.46 ± 9.68^*^	101.62 ± 7.10	101.08 ± 7.22
**SAB**	194.30 ± 9.66	204.76 ± 7.67^*^	189.84 ± 7.18	198.61 ± 7.59^*^	198.30 ± 5.39	198.77 ± 6.07
**SAD**	21.15 ± 3.15	25.83 ± 3.07^*^	23.46 ± 3.44	27.92 ± 4.72^*^	26.15 ± 4.45	26.00 ± 4.60
**TF**	28.30 ± 4.21	34.15 ± 4.46^*†^	24.92 ± 4.03	30.23 ± 3.98^*^	28.00 ± 3.91	28.08 ± 7.37
**TE**	23.76 ± 5.32	28.69 ± 5.58^*^	21.15 ± 10.08	25.31 ± 3.71^*^	25.07 ± 5.66	25.15 ± 5.55
**HF**	23.77 ± 6.48	29.15 ± 7.27^*^	23.15 ± 4.38	28.00 ± 4.55^*^	24.69 ± 7.59	25.08 ± 7.37
**HE**	132.54 ± 8.84	139.92 ± 7.83^*^	139.08 ± 6.74	143.92 ± 4.46^*^	136.38 ± 8.29	137.23 ± 8.65

* Significant difference from baseline;

† significant difference between G1 vs. CG and G2 vs. CG. Legends: (SF) shoulder flexion, (SE) shoulder extension, (SAB) shoulder abduction, (SAD) shoulder adduction, (TF) trunk flexion, (TE) trunk extension, (HF) hip flexion, (HE) hip extension.

**Table 3:** Effect sizes and improvement changes in flexibility measurements across 12 weeks of training.

	G1 (n = 13)	G2 (n = 13)	CG (n = 13)
**SF**	1.27	1.26	0.06
	Moderate	Moderate	Trivial
**SE**	1.24	0.77	0.08
	Moderate	Small	Trivial
**SAB**	1.08	1.22	0.09
	Moderate	Moderate	Trivial
**SAD**	1.48	1.08	0.03
	Moderate	Moderate	Trivial
**TF**	1.39	1.31	0.01
	Moderate	Moderate	Trivial
**TE**	0.92	1.09	0.01
	Moderate	Moderate	Trivial
**HF**	0.83	1.19	0.05
	Moderate	Moderate	Trivial
**HE**	0.83	0.71	0.1
	Moderate	Small	Trivial

Legends: (SF) shoulder flexion, (SE) shoulder extension, (SAB) shoulder abduction, (SAD) shoulder adduction, (TF) trunk flexion, (TE) trunk extension, (HF) hip flexion, (HE) hip extension.
